# Characterization and Performance of LbL-Coated Multibore Membranes: Zeta Potential, MWCO, Permeability and Sulfate Rejection

**DOI:** 10.3390/membranes10120412

**Published:** 2020-12-10

**Authors:** Saskia Dillmann, Shambhavi Arvind Kaushik, Jakob Stumme, Mathias Ernst

**Affiliations:** 1 Institute for Water Resources and Water Supply, Hamburg University of Technology, Am Schwarzenberg-Campus 3, 20173 Hamburg, Germany; mathias.ernst@tuhh.de; 2 DVGW Research Centre TUHH, Am Schwarzenberg-Campus 3, 20173 Hamburg, Germany; shambhavi.kaushik@tuhh.de (S.A.K.); jakob.stumme@tuhh.de (J.S.)

**Keywords:** LbL coating, characterization of capillary membranes, zeta potential measurement, MWCO determination, multibore hollow fibre membranes

## Abstract

The characterization of membranes is suitable to investigate changes in the membrane properties caused by Layer-by-Layer (LbL) modification. Besides permeability, rejection, and molecular-weight cut-off (MWCO), which give information about the modification of the separation behaviour of the membrane, the zeta potential is capable of describing the surface charge of the membrane and its variation impacted by the properties of the polyelectrolyte multilayers (PEM). In this study, a new method for zeta potential measurement of hollow fibre membranes with several capillaries was developed and further investigations on the LbL modification of such membranes were performed. The results showed that an LbL coating with 8 DL PDADMAC/PSS led to a significant increase in the membrane charge of more than 20 mV. The coating with a different number of polyelectrolyte (PE) layers showed a zig-zag behaviour, comparable to data from flat sheet studies. However, in contrast to most flat sheet membranes, the charge curve assumes a totally negative trajectory at neutral pH. Further experiments on the MWCO of the LbL-modified membrane showed a reduction in the pore diameter from approx. 20 nm to less than 2 nm, reaching the range of nanofiltration membranes. With information on both the zeta potential and the MWCO, it was found that the rejection mechanism in LbL-modified multibore membranes is a complex interplay between the sieving effect due to reduction in the pore diameter and the repulsion effect of the charged membrane.

## 1. Introduction

### LbL-Modification of Membranes

Commercial ultrafiltration (UF) membranes are increasingly being applied in advanced drinking water treatment to remove particles, turbidity, and pathogens. With a pore diameter between 10 and 20 nm, they are generally not suitable for the rejection of dissolved water constituents. Layer-by-Layer surface modification can be a powerful tool to modify porous membranes and consequently, their rejection behaviour, in such a way that retentions for certain dissolved water constituents such as sulfate, hardness, or dissolved organic matter can be achieved. During the LbL modification, the membrane is alternatingly coated with polycations and polyanions which adsorb on the charged membrane surface and form defect-free, very thin double layers [1]. This process can be repeated until the desired layer properties are reached. With increasing layer number, the separation limit of the coated UF membranes successively shifts into the range of nanofiltration (NF) membranes [2].

Compared to commercial NF membranes, the LbL-modified UF membranes might have some advantages: firstly, the preparation process of LbL-modified membranes is environmentally friendly when non-toxic polyelectrolytes (PEs) are used [3]. Secondly, the backwash ability of the UF membrane prepared by this process might be maintained [4,5]. Additionally, by controlling the defined coating conditions, it may be possible to tailor the LbL film to produce membranes with specific properties [6,7]. Compared to commercial NF membranes, operation at lower transmembrane pressures (TMP) may be possible as well as direct water treatment, without complex pre-treatment, leading to a more energy-efficient plant operation [8].

The conditions during the membrane coating are decisive for the structure of the polyelectrolyte multilayers and therefore, also for the resulting membrane properties. They can be influenced by the variation of coating parameters such as the number of double layers (DL), the molecular weight of the PE, temperature, pressure, pH value, or the background ionic strength in the coating solution. In coating solutions with low ionic strength, the PEs are stretched due to higher electrostatic repulsion forces; at high ionic strength, however, they tend to curl up, which leads to the formation of thicker, denser, and more stable layers [9,10]. Therefore, the ionic strength of the coating solution probably influences the thickness of the polyelectrolyte multilayers (PEMs) to a large extent.

The targeted application determines how many double layers are suitable for the LbL modification of the membranes. For example, Adusumilli and Bruening 2009 used 4–6 double layers of poly(styrenesulfonate) (PSS) and poly(diallyldimethyl ammonium chloride) (PDADMAC) for high SO_4_^−^ removal (90%) [11], whereas other researchers like Ji et al., 2008 worked with up to 60 bi-layers of polyethyleneimine (PEI) and polyacrylic acid (PAA) on hydrolysed polyacrylonitrile (PAN) ultrafiltration membranes to achieve separation of alcohol/water mixtures [12]. Certainly, the properties and porosity of the virgin membrane material are also decisive for resulting LbL properties. Another option to influence the polyelectrolyte multilayer membrane (PEMM) by coating conditions is the “dynamic adsorption” of the PEs [13]. By filtering the polyelectrolyte solution under pressure through the membrane pores, the larger pores are coated first and their pore diameters are reduced. This may result in a more uniform pore structure [12,14]. Moreover, coating under pressure can improve the backwash ability as the PEMs seem to be more stable [5].

The deposition of the polyelectrolytes leads to a change in the membrane properties. To determine how the membrane properties change due to the polyelectrolyte coating, common membrane characterization methods can be used, such as molecular weight cut-off, zeta potential, or contact angle. These methods not only show the change of the membrane properties due to the coating, but also give a hint as to how the ion rejection is influenced by the modification and how coating conditions might be optimized.

The MWCO is defined as the molecular weight of a substance that is 90% rejected by the membrane [15]. The MWCO consequently gives information about the pore size of the membrane, as all molecular weights higher than this value have a rejection of at least 90%. The MWCO changes drastically with LbL modification of a membrane. For ceramic hollow fibre membranes made from alumina and with a nominal pore size of 100 nm, a coating with six double layers of PDADMAC and PSS led to a MWCO of 240 Da [4]. A similar result of 250 Da was observed for UF hollow fibre PES membranes coated with 8 DL PDADMAC/PSS [2].

The zeta potential gives information on the surface charge of a membrane. As surface charge strongly influences the rejection of certain water compounds, the zeta potential is a crucial tool for membrane characterization to understand the rejection mechanism in LbL membranes. For LbL-modified membranes, a general zig-zag pattern is described, depending on the character of the final polyelectrolyte layer [11,16,17,18]. Depending on the virgin membrane material, the polyelectrolyte, and the coating conditions, the zeta potential behaves differently. PES flat sheet ultrafiltration membranes, coated with PDADMAC/PSS, showed a tendency progressing towards less negative zeta potential values with an increasing number of layers. Starting at 6 DL, the following layers had a positive zeta potential, independent of if a polycation or -anion was the terminating polyelectrolyte layer [11]. On the contrary, nanofiltration membranes, modified with PDADMAC/PSS, showed a clear shift between positive and negative zeta potential, consistent with the electrostatic orientation of the concluding polyelectrolyte. With the increasing number of layers, the amplitude expands, from about −50 mV at 4 DL to about −70 mV at 8 DL [16]. Su et al., 2012 showed that besides the nature of the virgin membrane and the polyelectrolytes, the coating procedure also plays an important role for the resulting zeta potential [13].

As natural dissolved organic water compounds are generally negatively charged, negatively charged membranes are usually used in water treatment in order to avoid membrane fouling. For LbL-modified membranes, not only does membrane fouling need to be avoided, but additionally, a negative charge may lead to a higher rejection of anions. In order to examine the charge of the modified membrane, to describe how it changes with an increasing number of layers, and to draw conclusions on the rejection behaviour of these membranes, it is of major importance to measure the surface charge with a reliable method. To the best of found knowledge, there is no measuring method for zeta potential in hollow fibre membranes with several capillaries (also called multibore membranes from hereon). As cutting the membrane to separate the capillaries would damage the membrane and probably destroy the LbL films, a measuring method was developed and tested to analyse the multibore membranes.

## 2. Materials and Methods

### 2.1. Materials

Poly(diallyldimethyl ammonium chloride) (PDADMAC; Mw = 400,000–500,000 g/mol, 20 wt % solution), poly(styrenesulfonate) sodium salt (PSS; Mw = 1,000,000 g/mol, powder), and dextran standards of 70,000, 100,000, and 150,000 Da were purchased from Sigma Aldrich (Taufkirchen, Germany). Sodium chloride (NaCl; Mw = 56.488 g/mol, powder) and magnesium sulfate (MgSO_4_∙7H_2_O; Mw = 120.366 g/mol, powder) were purchased from Carl Roth (Karlsruhe, Germany). Polyethylene glycol (PEG) standards of 200, 300, 400, 600, 1000, 1500, 2000, 6000, 10,000, 20,000, and 35,000 Da were purchased from Merck KGaA (Darmstadt, Germany). All solutions were prepared in deionized water (Milli-Q, Millipore Corporation, Billerica, MA, USA).

### 2.2. Membrane

The experiments were carried out on Multibore^®^ membranes (INGE GmbH/Dupont, Greifenberg, Germany), a capillary polyethersulfone (PES) membrane operated inside out. The ultrafiltration membrane consists of seven single capillaries, each with a diameter of 1.5 mm (MB 1.5) or 0.9 mm (MB 0.9), in one membrane fibre. The capillaries are arranged in a honeycomb structure, therefore having a very high stability. The membrane has an active layer of negatively charged PES and a MWCO of approx. 100,000 Da (manufacturer information).

The membranes used in the experiments were lab modules of 25–30 cm, which conforms to a membrane surface of approx. 0.006 m^2^. The membranes were flushed with at least 1 L deionized water (Milli-Q, Millipore Corporation, Billerica, MA, USA) before coating to remove all residues from the membrane.

### 2.3. Coating

**Figure 1:**
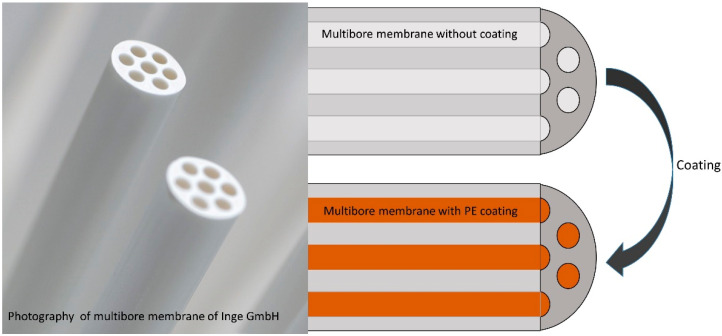
Coating of multibore membrane with varying layers of polyelectrolytes inside the capillaries (image: [19]).

For the LbL modification, the membrane was coated with cationic and anionic polyelectrolytes inside the capillaries, as shown in Figure 1.

The polycation PDADMAC and the polyanion PSS were used for the coating process, both known to be stable against acids, alkalis, and oxidants which are used for the usual cleaning of ultrafiltration membranes [4,20]. PE solutions were prepared in solutions containing a concentration of 0.1 M NaCl background ions. As the membrane is negatively charged, first PDADMAC is deposited on the membrane surface with a coating time of 3 min. After a washing step with pure water to remove the excess PDADMAC, the PSS coating followed also with a contact time of 3 min. The coating with these two PEs is called one double layer and can be repeated as often as necessary to achieve the desired properties.

For the coating of the ultrafiltration membranes, a coating machine, the Nanocoater (Surflay Nanotec, Berlin, Germany), was used. The coater is computer-controlled to achieve a uniform and reproducible coating on the membrane surface.

**Figure 2:**
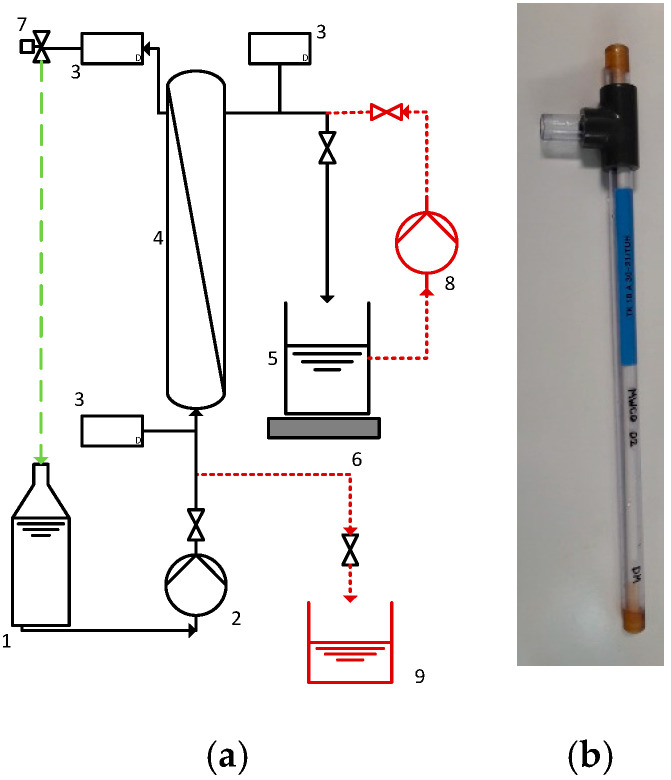
Scheme of the filtration plant (**a**) and the membrane module (**b**).

The lab modules of the capillary membranes (Figure 2b) were directly connected to the Nanocoater. The flat sheet membranes were coated in a self-constructed cell that was also connected to the Nanocoater.

### 2.4. Filtration Setup and Experiments

The filtration experiments were performed with a lab-scale filtration plant (Figure 2a) for capillary membranes (Figure 2b). The membrane (4) was potted in an acrylic pipe with a feed inlet, a permeate, and a retentate outlet so that it could be operated in dead-end as well as cross-flow mode. The permeate was collected on a scale (5, 6), so the flux and permeability could be determined. Three pressure gauges (3) were placed to determine the TMP and a control valve (7) was used to regulate the stream, the recirculation, and the TMP.

All experiments were implemented in cross-flow mode at a cross-flow velocity of 0.03 m/s and a yield of 25%. Depending on the layer number, the plant was operated at an increasing TMP, starting at 0.2 bar at layer no. 1 up to a maximum pressure of 2.5 bar for 5–8 DL. For filtration experiments regarding the rejection behaviour of the membranes, a 100 mg/L sulfate solution of magnesium sulfate was used. For the determination of the MWCO, 1 g/L solutions of PEG and dextran standards were used.

### 2.5. Characterization Methods

In addition to the pure water permeability and sulfate rejection rates, membrane material properties such as the molecular weight cut-off [21] and the zeta potential were determined [22]. Moreover, scanning electron microscope (SEM) pictures were taken to show the morphology of the membranes.

The permeability and rejection were determined with MB1.5 membranes. Three membranes were coated with double layers of PDADMAC/PSS. Subsequently, the permeability and sulfate rejection was tested in the filtration plant. The separation behaviour of the modified membranes was tested by the determination of the rejection of divalent ions, SO_4_^2−^ from MgSO_4_∙7H_2_O. The sulfate concentration in the feed and permeate was measured using the photometric measurement after the precipitation reaction with barium chloride (referring to the Hach Lange Method 8051 [23]).

### 2.6. MWCO

The molecular weight cut-off is one of the most commonly used methods for membrane characterization. According to Crittenden et al., 2012 [24], the pore diameter can be calculated by Equation (1) using the MWCO.

*d*_*pore*_ = 0.11∙*M*^0.46^ (1)

*d_pore_* = hydraulic pore diameter, nm; *M* = molecular weight according to MWCO, g/mol.

The determination of the MWCO was done with polyethylene glycol (PEG) and dextran standards of different molecular weights, which were used in a mixture of 1 g/L for each substance as the feed solution. The feed and permeate samples were analysed with gel permeation chromatography with an RI detector.

### 2.7. SEM Imaging

The SEM pictures were taken at the “central service unit SEM” of the Hamburg University of Technology. The membrane samples were sputtered with a 5 nm gold layer and analysed in the scanning electron microscope Zeiss Supra 55 VP FEG-SEM.

### 2.8. Zeta Potential—Measurement of Flat Sheet Membranes

The zeta potential, which arises as an electrical double layer developed at the solid–liquid interface, describes the surface charge of a membrane. It is influenced by the functional groups on the membrane surface, the ions in the feed solution, and the pH. The zeta potential for capillaries can be described by an approximation of the Helmholtz–Smoluchowski equation [22].

For zeta potential measurements, a SurPASS Electrokinetic Analyzer (Anton Paar GmbH, Graz, Austria) was used and adapted. The flat sheet membranes were prepared for the measurement by storing it in pure water for 24 h to compensate the swelling of the membrane and to remove residual substances. It was then fixed on the stamps of the measuring cell and connected with the measuring electrodes. The gap between the two membranes was adjusted to 100 µm in order to be able to apply the needed pressure gradient. The measurement was performed at 1 mmol/L KCl, starting at pH 9. After the cell was washed by the electrolyte, the zeta potential was determined at different pH values between 9 and 3, by titration of HCl. The zeta potential was calculated with the software Attract (supplied by Anton Paar) based on the Helmholtz–Smoluchowski equation. To compare the results, the capillary and flat sheet membranes both were measured by the Streaming Potential method.

## 3. Results and Discussion

### 3.1. Zeta Potential—Innovative Measurement Method for Multibore Membranes

The zeta potential can be used to describe the status of the charge of surfaces, including uncoated and PDADMAC/PSS-modified membranes. In hollow fibre membranes with several capillaries, the measurement is difficult as the measurement device is usually designed for the determination of single hollow fibres with a limited outer diameter [25]. The major limitation for the multibore system was the achievement of a pressure gradient that is necessary for the zeta measurement. While flat sheet membranes are measured in a cell with a 100 µm gap between the membranes, single bore membranes cannot have an outer diameter larger than 2 mm since the needed pressure gradient cannot be reached and the measurement is then aborted. For the multibore membrane, which has an outer diameter of 4 mm, a sufficient pressure gradient was needed over all seven capillaries of the membrane.

It was thus required to make physical changes to the measuring cell for single bore membranes. More precisely, the length and the diameter of the cell had to be adapted, as several trials showed that a too short or even a too long piece of membrane led to a termination in the measurement. Several trials revealed that only at a length of 8 cm, it was possible to reach a pressure of 200 mbar in uniform gradual steps and ensure a successful measurement.

**Figure 3:**
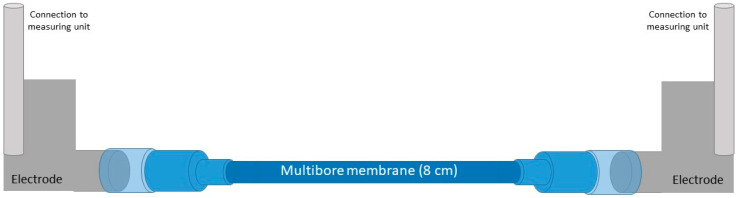
Experimental setup for measuring zeta potential of multibore membranes.

An additional problem occurred for the uncoated and first 2 DL coated multibore membranes. The pressure of 200 mbar already led to a permeation of the solution through the membrane. After a certain time of measurement, the pressure in the system decreased due to the solution permeation, leading to a pressure loss and in consequence, to a termination of the whole measurement. To overcome this challenge, various options of sealing the outside surface of the membrane were tested. The use of different glues as well as shrinking tubes were tried in vain, as the measurement was either aborted or did not show a constant and reliable result. In the end, a tube was used whose inner diameter conforms to the outer diameter of the membrane. Two flexible pipes were used to connect the membrane to the electrodes. These adjustments prevented leakages and a stable and plausible measurement was achieved. A scheme of the new constructed setup is shown in Figure 3.

With these fresh adaptations to the measuring system, measurements of the zeta potential in multibore membranes were successfully performed.

### 3.2. Zeta Potential of Multibore Membranes

**Figure 4:**
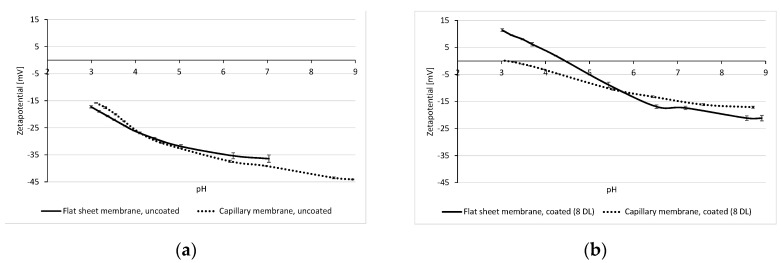
Zeta potential measurement for uncoated (**a**) and 8 DL-coated (**b**) capillary and flat sheet membranes.

To validate the zeta potential measurements, values of the multibore membranes were compared with the results of the flat sheet PES membrane UP 150 from Microdyn-Nadir (Wiesbaden, Germany). Due to the same material (PES) and similar MWCO/pore size situation (approx. 150 kDa), a comparable zeta potential of both membranes was expected. Results of the measurements are displayed in Figure 4. The graph of the non-coated multibore membrane is in very good agreement with that of the flat sheet membrane. Both PES membranes exhibit a zeta potential of approx. −18 mV at a pH of 3, decreasing similarly with increasing pH. The results were quite reproducible, leading to the conclusion that the adaption of the method to measure the zeta potential of hollow fibre membranes with several capillaries was successfully adapted.

The results of the zeta potential measurement of the membranes coated with 8 DL of PDADMAC/PSS are also in good agreement (Figure 4b). The coated flat sheet as well as multibore membrane have a negative zeta potential of approx. −18 mV at pH 8.5, which then decreases (becomes less negative) with decreasing pH. For the multibore membrane, the isoelectric point (IEP) is reached at pH 3, while the flat sheet membrane reaches it at a pH of 4.5. These discrepancies in IEPs might be due to the effect of layer defects due to an inhomogeneous coating in the self-constructed test-cell. The inflow of the PE solution into the planar coating cell through a single tube may not have fully ensured plug flow conditions of the coating solutions and might have resulted in non-ideal homogeneous coating over the membrane surface.

**Figure 5:**
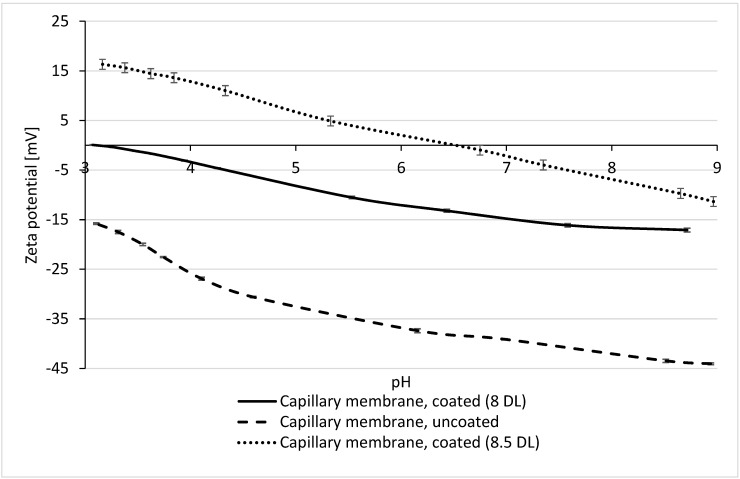
Zeta potential of uncoated (dashed), 8 DL- (line), and 8.5 DL- (dotted) coated membranes.

After the successful adaptation of the measuring method, the effect of the LbL coating on the multibore membranes with regard to the zeta potential was investigated in more detail (Figure 5).

The zeta potential of the virgin PES multibore membrane is negative over the total pH range: −43 mV at pH 9 and −16 mV at pH 3, due to the negative charge of the PES material of the active membrane layer. After coating with 8 DL of PDADMAC/PSS, it shifts to less negative values, starting at −17 mV at pH 9 and reaching the isoelectric point at pH 3.1. One would expect that the membrane coated with negatively charged PSS as the terminating layer would have a more negative zeta potential. The increasing zeta potential with decreasing pH is attributed to the dissociation of acidic functional groups—at lower pH, the concentration of H_3_O^+^ becomes more dominant and the surface starts assuming a positive charge [23]. However, here, the shift in zeta potential to less negative values for 8 DL membranes as compared to the virgin membrane was, in fact, a consequence of the increased number of PE layers.

For the 8.5 DL coated membrane with the positively charged PDADMAC as the top layer, it was expected that the zeta potential would be rather positive over the entire pH range. As shown in Figure 5, however, the zeta potential starts with a negative value of −11 mV at pH 9, reaches the IEP at a pH of 6.7, and increases up to 16 mV at pH 3. Although the zeta potential is positive below pH 6.7, it may be possible that at higher pH, the influence of the negative charge of the membrane is stronger and therefore, partly shields the positively charged functional groups of PDADMAC [13].

**Figure 6:**
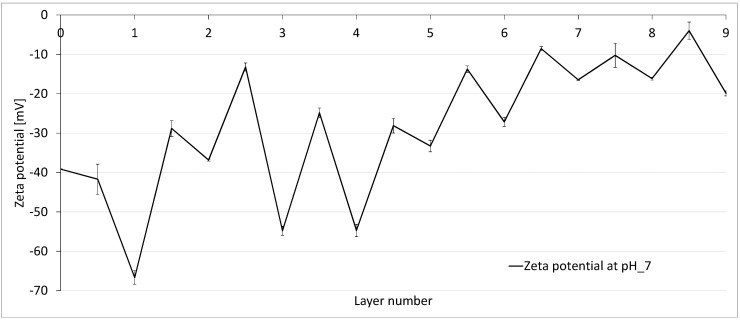
Zeta potential measurements of each layer of LbL-modified multibore membrane.

In order to obtain a better understanding of the electrostatic behaviour of the virgin multibore membrane and how it changes by polyelectrolyte coating, the development of zeta potential with each layer of PES was investigated. The zeta potential of the PSS terminated layers was expected to be negative as it is a negatively charged polyelectrolyte. Conversely, it was expected that the PDADMAC terminated layer would have a rather positive zeta potential due to the positively charged PE. In Figure 6, the values for zeta potential at ca. pH 7 for every single layer are displayed.

The curve behaves in a zig-zag pattern, depending on the terminating layer, which is a known effect from the literature data of flat sheet membranes [11,16,17]. The PSS terminating layers have, in general, a more negative value compared to the PDADMAC terminated layers. PDAPMAC terminated layers exhibit a higher zeta potential (less negative) albeit with a negative sign over the whole pH range. In layer numbers 1–4, it can be seen that the PSS terminated layers are in the range of the virgin membrane or even have a more negative value (e.g., −36 mV at 2 DL and −54 mV at 3 DL). This effect can be attributed to the contribution of the negative charge of the polyelectrolyte. From layer number 5, the more positive/less negative trend of the zeta potential is apparent, as the zeta potential reaches a value of −16 mV at 8 DL.

Compared to the PSS terminated layers, zeta potential values for the PDADMAC terminated layers were expected to be positive due to the positively charged PDADMAC as the uppermost layer. However, as displayed in Figure 6, in a neutral pH range of pH 7, the membrane possessed a negative zeta potential over all layers. This effect can be explained by the effect that the negative charge of the membrane and negatively charged PSS are partly shielding the positively charged amino groups of the PDADMAC, leading to a total negative zeta potential [13]. Only in the acidic range, at pH 3, the zeta potential became positive after layer number 5.5 and showed increasingly positive potentials for the following layers.

Despite the negative zeta potential at a pH of 7 over all layers, an increasing trend in the zeta values can be seen. The zeta potential shifts to a less negative range from −67 mV at 1 DL to −16 mV at 8 DL, as more layers are deposited on the membrane. The zig-zag effect is known from the literature, e.g., reported by Reurink et al., 2018 [18] in hollow fibre membranes as well as Adusumilli and Bruening 2009 [11], where PES flat sheet membranes were coated with PSS and PDADMAC. The zeta potential of the membranes was positive for all PDADMAC terminated layers and also became positive even for PSS terminated layers, after the deposition of 6 DL. With further experimental methods, Adusumilli and Bruening found that a higher amount of PDADMAC enters the layers, compared to PSS, and tends to migrate to the surface. Thus, after a certain number of layers, when PSS is further deposited, PDADMAC forms complexes with the PSS, yielding a more positive potential [11]. The contrary effect was observed by Malaisamy et al., 2011 for PDADMAC/PSS-modified NF270 membranes—the zig-zag variation of the zeta potential with each layer increases in magnitude as the number of layers increases; i.e., PSS terminated membranes are more negative and PDADMAC terminated membranes are more positive at higher number of layers [16].

However, differences in the coating procedure as well as the membrane geometry may also influence the zeta potential. As described in Section 2.3, the multibore membranes in this study were coated with the Nanocoater (Surflay Nanotec, Berlin, Germany), which ensures a proper and defined coating of capillary membranes and its active separation layer. It is likely that the charge of the supporting structure also has an impact on the zeta potential, as the surface is comparably larger than the surface of the active layer. It is possible that the negative charge of the supporting structure of the multibore membranes used in this work influences the measurement, leading to the same zig-zag pattern as known in the literature, but with a shift to the negative range. Additionally, specifically in the case of multibore membranes, the supporting structure of one capillary is not the only one influencing the charge; rather, the whole supporting structure around all the seven capillaries contributes to the overall charging behaviour of the membrane. Su et al. 2012 showed that different methods in depositing the polyelectrolyte layers on a polysulfone (PS) membrane result in very different zeta potential results. When the membrane was coated in a static assembly, one layer of PDADMAC resulted in a negative zeta potential, due to the stronger negative charge of the PS membrane, shielding some of the positively charged amino groups of the PDADMAC. In contrast, with a dynamic assembly, filtering the polyelectrolyte solution in cross-flow mode through the membrane, and thereby also coating the support structure of the membrane, the zeta potential became +55 mV [13]. These findings support the theory that the zeta potential is strongly impacted by the charge of the support layer and the overall coating procedure.

### 3.3. Influence of Zeta Potential on the Permeability and Rejection

**Figure 7:**
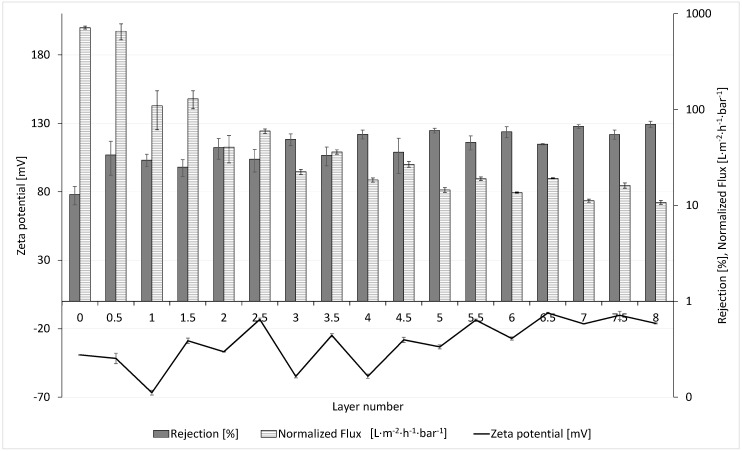
Normalized flux, sulfate rejection, and zeta potential of LbL-modified multibore membranes vs. number of PE layers (PDADMAC/PSS).

As shown in the previous section, the surface charge of the membrane changes drastically due to the LbL coating. The influence of zeta potential on the normalized flux (flux normalized to one bar) and sulfate rejection is displayed in Figure 7. The mean values of three membranes and the standard deviation are shown for the development of the normalized flux and sulfate rejection, with increasing number of layers.

According to the manufacturer, the pure water permeability of the membrane is 1000 L∙m^−2^∙h^−1^∙bar^−1^. In our filtration plant, a pure water permeability of 720 L∙m^−2^∙h^−1^∙bar^−1^ was reached.

The data of the normalized flux as well as the rejection show a zig-zag pattern in accordance with the data of the zeta potential. In general, the flux, starting with more than 700 L∙m^−2^∙h^−1^∙bar^−1^ for the uncoated membrane, diminishes after coating of the first two layers by nearly 95% to a value of 40 L∙m^−2^∙h^−1^∙bar^−1^. In the following six layers, the decrease is less severe, but nevertheless, it decreases continuously until it reaches a minimum value of approx. 12 L∙m^−2^∙h^−1^∙bar^−1^ at 8 DL. Simultaneously, along with the normalized flux, the rejection of the divalent ion, sulfate, increases nearly linearly with each DL (PSS terminating) up to 70% at 8 DL. It is apparent that the PEMs retain the sulfate ions, but simultaneously cause an additional hydraulic membrane resistance. This is in agreement with the literature data [26,27] of flat sheet and hollow fibre membranes, where, depending on the coating conditions, approx. 75% [27] and up to 96% [26] sulfate rejection were reached. De Grooth et al. 2015 also explained the development of permeability by two different effect—the pore-dominating and the layer-dominating regime [26]. Due to a drastic drop in the permeability, it seems apparent that the coating of the membrane starts with attachment of polyelectrolytes in the membrane pores in the first two layers, also called the pore-dominated regime, leading to an abrupt decline in the permeability. Thereafter, it is probable that the coating continues on top of the membrane pore structure, called the layer-dominated regime, leading to a less steep decline in the permeability. It is likely that the PEMs form an additional, nearly dense membrane on top of the underlying UF membrane, which causes a further decrease in the permeability and the continuous increase in rejection, subsequently leading to membrane properties that correspond to NF membranes [21]. Even though the PE layers on the membrane are not cross-linked, initial lab scale experiments with the 8 DL-coated membrane show a good stability during backwash. The permeability stayed nearly constant over 10 backwash and filtration cycles at a backwash pressure of 2.5 bar for 60 s (data not shown). Pilot experiments in a waterworks are planned to investigate PEM long-term stability under higher cross-flow velocities, different backwash fluxes, and use of chemical cleaning agents.

The PDADMAC terminated layers generally show higher permeabilities compared to the previous PSS terminated layer. It is known from the literature [10,28,29] that PDADMAC has a much higher swelling behaviour than PSS. The polymer film swells more when PDADMAC is the terminating layer, leading to thicker but less dense layers. This swelling effect may lead to a higher membrane permeability [13]. Additionally, McCormick et al. [30] observed that water is more mobile within the PE film in PDADMAC terminated membranes than in PSS terminated membranes. The reason for this is still not solved.

As for the phenomenon of the simultaneous lower rejection of sulfate for the PDADMAC terminated membranes, there are two possible explanations. On the one hand, it is possible that the rejection is constant, but due to dilution, because of the higher permeability, the average rejection drops. On the other hand, the loose structure of the layers may lead to lower rejection rates for sulfate ions compared to the membranes with the PSS terminated coating. Furthermore, it can be suggested that repulsive forces of the negatively charged PSS layer and the SO_4_^2−^ ion are responsible for the higher rejection of the PSS terminating membranes.

### 3.4. SEM Analysis of Coated and Uncoated Membranes

**Figure 8:**
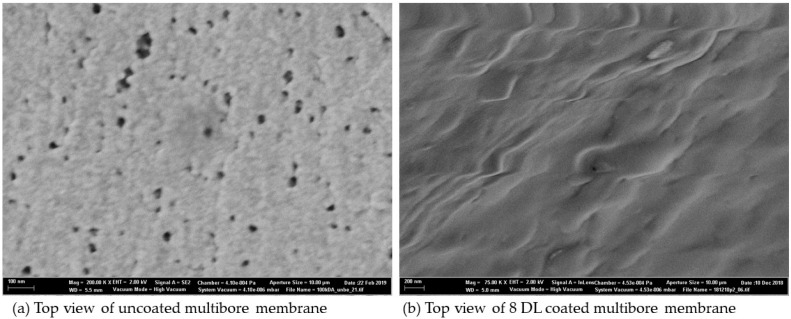
SEM pictures of uncoated (**a**) and 8 DL-coated (**b**) multibore membranes.

SEM pictures of the membrane give additional information on how the morphology of the membrane is changed by the polyelectrolyte coating. In the following figure, pictures of the top view of the uncoated and 8 DL-coated multibore membrane are presented (Figure 8).

Top view pictures of the membranes are considered in high resolution to show the effect of the coating on top of the membrane surface. In Figure 8a, the uncoated membrane can be seen with pores in a range of 10–30 nm; after LbL coating with 8 DL (Figure 8b), the surface becomes smooth and no pores are visible anymore. This effect supports the above-mentioned theory that the PEMs form an additional dense structure on top of the underlying membrane.

### 3.5. Determination of the Molecular Weight Cut-Off

**Figure 9:**
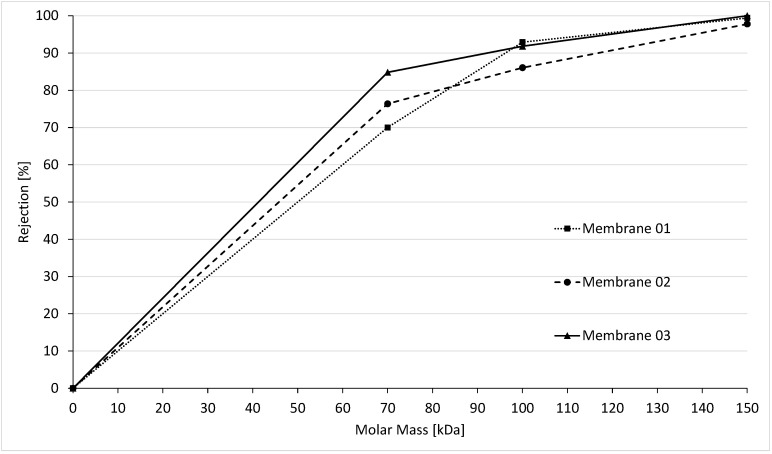
Molecular weight cut-off for uncoated PES multibore membranes.

To determine the MWCO, the rejection of different molecular weight substances that were filtered through the membrane was measured. Consequently, based on PEG and dextran rejection, it was possible to obtain an MWCO curve for the uncoated and coated membranes. According to the manufacturer information (Inge GmbH/Dupont, Greifenberg, Germany), the MWCO of uncoated Multibore membranes should be at 100,000 Da. Investigating the MWCO value based on the experimental procedure previously described resulted in an average cut-off value of 101,000 Da for the three membranes tested, as shown in Figure 9.

The average value is in very good accordance with the manufacturer data. In the single experiments, it became apparent that the individual uncoated membranes differ in a range of 92,000, 96,000, and 116,000 Da. This might be a result of the very small lab modules used in the experiments which only had a surface of <0.01 m^2^ compared to commercially used membrane modules of several m^2^.

**Figure 10:**
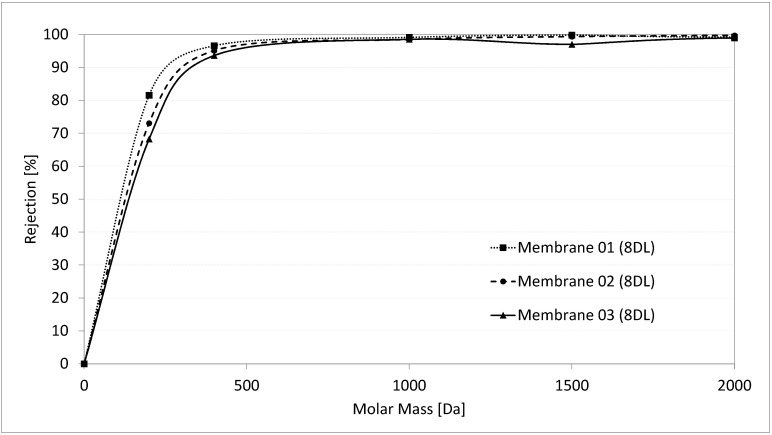
Molecular weight cut-off for 8 DL-coated multibore membranes.

After the coating with 8 DL of PDADMAC/PSS, the mean MWCO of three membranes became 335 Da, with values of 300, 350, and 360 Da, displayed in Figure 10. This means that the 8 DL coating leads to a reduction in the MWCO of the membrane by more than 99%. These values are consistent with the results of Menne 2017, who also determined the MWCO of modified hollow fibre PES membranes and measured a mean value of 250 Da for 8 DL coating of PDADMAC/PSS (applied in similar coating conditions, 0.1 M of NaCl) [2].

The pore diameter of the coated membranes was calculated according to Crittenden et al., 2012 [24], using the molar mass, resulting in a value of 1.6 nm for the 8 DL-modified membrane. This value indicates a membrane with a pore diameter in the range of a nanofiltration membrane [15]. The permeability data for the modified membranes are in agreement with this calculation, showing results in the range of nanofiltration membranes. However, the rejection of divalent ions (sulfate) is much lower compared to commercial nanofiltration membranes, which have a rejection of >95% [15]. This supports the assumption that the rejection mechanism in LbL membranes is not only dependent on the size exclusion but also on the membrane charge.

The results of the zeta potential measurements and the MWCO determination suggest that both the decrease in the pore diameter as well as the change in the surface charge have a major impact on the rejection behaviour. In general, a higher rejection can be associated with a more negative zeta potential, but simultaneously, the membrane has a lower permeability. A less negative zeta potential leads to a lower average rejection of divalent ions. Therefore, PSS terminated membranes generally show a better rejection (compared to PDADMAC terminated membranes) due to their negative zeta potential, but at the same time, have a lower permeability. The increasing zeta potential with the rising number of layers leads to the assumption that the positive charge of the polycation PDADMAC leads to less rejection of sulfate. It is probable that the 70% rejection for 8 DL-modified membrane is mainly a result of the reduction in pore size to 1.6 nm. This assumption is also supported by further rejection experiments at pH 3. As shown in Figure 4, at pH 3, the IEP is reached, where the net charge of the membrane is zero. Filtration of an 8 DL-coated membrane at this pH shows a decline in the sulfate rejection to 30%, compared to 70% at pH 6. Although the rejection of 30% could also probably be reached due to the smaller pore size, it is obvious that the zeta potential has a huge impact on the rejection of the divalent ions, as even a slightly negative zeta potential of −15 mV at pH 6 increases the rejection by 40%.

## 4. Conclusions

In this paper, we present the successful adaption and implementation of the zeta potential measurement in multibore membranes. With this innovative method adaption, it was possible to determine the zeta potential in hollow fibre membranes with several capillaries and to show how the zeta potential is altered due to the deposition of PEM. The zeta potential changed depending on the terminating layer of PSS or PDADMAC, leading to a zig-zag pattern. This zig-zag behaviour was also found for the permeability, as well as for the rejection, of a divalent ion (sulfate), showing that a more negative zeta potential can be associated with a higher rejection, while a less negative zeta potential leads to a lower rejection. Additionally, a general trend of an increase in the total values of the zeta potential was observed. This phenomenon could be explained by the more mobile PDADMAC, which has a higher swelling capacity, leading to a migration of the polycation to the surface of the membrane and thus, causing an increased zeta potential.

Additional investigations on the MWCO have shown that the pore size of the 8 DL modified membrane is in the range of a nanofiltration. As the permeability data support this determination, the rejection, which was at 70% for an 8 DL coated membrane, did not reach the value of NF membranes, which usually has a sulfate retention >95%. Evidently, the rejection is not only dependent on the smaller pore size of the modified membrane, but is also strongly influenced by the less negative zeta potential at 8 DL.
